# Latency-associated upregulation of SERBP1 is important for the recruitment of transcriptional repressors to the viral major immediate early promoter of human cytomegalovirus during latent carriage

**DOI:** 10.3389/fmicb.2022.999290

**Published:** 2022-11-24

**Authors:** Emma Poole, John Sinclair

**Affiliations:** Department of Medicine, University of Cambridge, Cambridge, United Kingdom

**Keywords:** human cytomegalovirus, latency, CHD3, SERBP1, KAP1

## Abstract

Suppression of human cytomegalovirus (HCMV) major immediate early gene (IE) expression from the viral major immediate early promoter (MIEP) is known to be crucial for the establishment and maintenance of HCMV latency in myeloid progenitor cells and their undifferentiated derivatives. This suppression of the MIEP during latent infection is known to result from epigenetic histone modification imparting a repressive chromatin structure around the MIEP in undifferentiated myeloid cells. In contrast, reactivation, resulting from, e.g., myeloid cell differentiation, is associated with activatory chromatin marks around the MIEP. Recently, recruitment of the transcriptional repressor SETDB1, *via* KAP1, to latent HCMV genomes was shown to be involved in latency-associated MIEP suppression in CD34+ progenitor cells. KAP1 is also known to associate with Chromodomain-helicase-DNA-binding protein 3 (CHD3) as part of the NuRD complex which can aid transcriptional silencing. We now show that the cellular protein Plasminogen activator inhibitor 1 RNA-binding protein (SERBP1), a known interactor of CHD3, is significantly upregulated during HCMV latency and that this protein is required for MIEP suppression during latent infection of myeloid cells. We further show that SERBP1 mediates CHD3 association with the MIEP as well as KAP1 association with viral genomic DNA. We suggest that SERBP1 functions as a scaffold protein to recruit transcriptional repressors to the latent viral genome and to mediate transcriptional silencing of the MIEP during latent carriage.

## Introduction

HCMV is the prototypic beta-herpesvirus which is usually asymptomatic upon primary infection but establishes a lifelong infection which is tightly controlled by the host immune system. In contrast, HCMV causes significant disease in the absence of a functioning immune system such as in those individuals undergoing immunosuppression during organ transplantation or in immunonaive neonates. The reason that HCMV infection is never cleared from the infected individual but maintained for life is, at least partly, due to its ability to establish a “latent” infection whereby cells carry viral genome in the absence of production of infectious virions but in a form that can be reactivated (Poole et al., 2014). For HCMV, it is known that one site of latency *in vivo* is in undifferentiated cells of the myeloid lineage, in particular CD34+ bone marrow progenitor cells and their monocyte derivatives (Sinclair and Poole, 2014). However, once these cells differentiate to macrophages or dendritic cells (DCs), virus sporadically reactivates from latency and virions are produced. In the immunocompetent, such sporadic reactivation is well controlled by the host immune response and is sub-clinical. In the immunocompromised, however, such reactivation events are not controlled and lead to virus dissemination and lytic infection in multiple cell types.

In lytic infection, a temporal cascade of viral gene expression occurs starting with immediate early (IE) gene expression, driven from the viral major IE promoter/enhancer (MIEP), which is key to driving subsequent expression of early (E) and then late (L) viral genes. In contrast, during latent infection, suppression of the viral MIEP occurs which is associated with histone marks of transcriptional repression around the MIEP; in contrast to histone marks of transcriptional activation which are present on the MIEP during reactivation (Sinclair and Poole, 2014). Although a plethora of cellular and viral factors have been identified which negatively or positively regulate the MIEP in a differentiation-dependent manner, how these are orchestrated to control HCMV latency and reactivation is still far from clear. One of the cellular factors known to be involved in MIEP suppression during latency is the Krüppel-associated box (KRAB)-associated protein 1 (KAP1/TRIM28) which has been shown to be recruited to the viral genome in latently infected myeloid cells and phosphorylation of KAP1 appears to act as a switch between latency and reactivation (Rauwel et al., 2015).

Upon chromatin tethering, KAP1 is known to scaffold epigenetic repressive components such as Histone 3 Lysine 9 (H3K9) methyltransferases (e.g., SETDB1; Schultz et al., 2002), Heterochromatin-Protein 1 (HP1) proteins (Nielsen et al., 1999; Ryan et al., 1999) as well as the NuRD histone deacetylase complex (Schultz et al., 2001, 2002), to promote chromatin condensation and transcriptional repression. Members of the NuRD complex, include CHD3 and CHD4 and phosphorylation of KAP1, which prevents KAP1-mediated repression of the HCMV MIEP, is known to cause CHD3/4 to be released from the repressive NuRD complex (Goodarzi et al., 2011) which can result in transcriptional activation (Hoffmeister et al., 2017). Thus, the presence of CHD3 and 4 is often associated with a transcriptional repression.

However, while the NuRD complex is generally associated with transcriptional repression, it is known that CHD3 and CHD4 can also have transcriptional activation properties (Hoffmeister et al., 2017). Indeed, during DNA damage repair, CHD4 and CHD3 act antagonistically to mediate activatory and repressive complexes, respectively. Therefore, CHD4 recruitment to the NuRD complex occurs when Kap1 is phosphorylated and repressive CHD3 is removed (Goodarzi et al., 2011). Interestingly, during lytic infection, it is known that viral pUL38 and pUL29/28 interact with CHD4 NuRD complexes to enhance IE accumulation (Terhune et al., 2010). Thus, it is possible that a balance of CHD3 and CHD4 recruitment to the NuRD complex could act to regulate the latent/lytic switch. While is it known that lytic viral genes are required to recruit CHD4-containing NuRD complexes during lytic infection to activate the MIEP in permissive cells, it is not known if CHD3 or 4 are recruited to viral genomes during latency, and, if so, how this may affect latency.

One known binding partner of CHD3 is SERBP1 (Lemos et al., 2003) which suggested that SERBP1 may play a role in regulation of the NuRD complex. Additionally, SERBP1 has been shown to maintain high levels of methionine in the cell which may also be needed for repressive histone modification (Kosti et al., 2020). Both activities of SERBP1 could lend themselves to functions which might be predicted to be required for the maintenance of a latent infection. Here we show that SERBP1 is significantly upregulated during HCMV latency and that, without SERBP1, the MIEP is active in otherwise latently infected cells. Furthermore, our observations show that, in the absence of SERBP1, the HCMV genome is not associated with either KAP1 or CHD3, suggesting that SERBP1 may act as a scaffold protein to enhance a repressive chromatin structure to aid the maintenance of HCMV latency.

## Materials and methods

### Cells and viruses

Primary monocytes were isolated from apheresis cones or venous blood as described previously (Aslam et al., 2019). HFFF, THP1, and 293 T cells were obtained from ATCC and maintained as previously (Poole et al., 2021).Two HCMV TB40E-derived viruses were used which have been described previously: TB40E-IE2YFP, which expresses YFP fused to the immediate early gene IE2 (IE86) and TB40E-GATA2mCherry which expresses mCherry from the GATA2 promoter (Elder et al., 2019; Poole et al., 2019). Lentivirus to generate shRNA SERBP1KD cells was obtained from Santa Cruz.

### Proteomic screen

The original unbiased proteomic screen identifying the upregulation of SERBP1 during latent HCMV carriage in monocytes has been published in full (Aslam et al., 2019).

### Western blot

Cell lysates were analyzed by Western blotting using the following primary antibodies: Anti-actin rabbit polyclonal (Abcam), anti-SERBP1 (Abcam) followed by the appropriate horseradish peroxidase (HRP)-conjugated secondary antibody, and analyzed by chemiluminescence.

### Immunofluorescence

Adherent cells were fixed with 4% paraformaldehyde for 20 min before permeabilization with 0.5% Triton X-100 for 20 min and blocking in 3% bovine serum albumin (BSA)/PBS for 1 h. After this time, primary antibody was added. Anti-SERBP1 primary antibody was added (1 in 200 dilution) for 1 h at RT and, after washing, cells were then incubated with the relevant Alexa Fluor 488 secondary antibody with Hoechst 33342 for 1 h before visualization by fluorescence microscopy.

### Chromatin immunoprecipitation

Chromatin immunoprecipitations (ChIPs) were carried out using the Imprint ChIP kit (Sigma) with antibodies anti-histone H3 (Upstate), anti-dimethylated (K9) histone H3 (Upstate), anti-acetylated (K8) histone H4 (Invitrogen) using the manufacturer protocol and as described previously (Poole et al., 2021).

### RTqPCR

RT-qPCR analyses were carried out using the QuantiTect (Qiagen) SYBR kit using standard primers and parameters for glyceraldehyde-3-phosphate dehydrogenase (GAPDH) and specific IE exon2/3 and UL138 primers previously described (Poole et al., 2021).

### Droplet PCR

Genome copy number was assessed using the Biorad QX200 system as previously described (Poole et al., 2021).

### shSERBP1 KD cell line

The lentiviral expression vector encoding SERBP1shRNA and puromycin resistance (Santa Cruz Biotechnology).To generate lentiviral particles, 293 T cells were seeded into 6-well plates at 5×105 cells/well were transfected into 293 T cells using transfection TRANSit 293 (Mirus) according to the manufacturer’s instructions. 24 h post transduction, media was replaced with 2.5 ml RPMI supplemented with 30% fetal calf serum. 24 h post media change, media was harvested containing lentivirus. This virus was used to inoculate 2.5 × 105 THP-1 cells which were pelleted, and then resuspended in the lentiviral or control supernatant in a 6-well plate. Polybrene was added to the cells at 2 μg/ml and the cells were then centrifuged in the plate at 600 xg for 45 min, and incubated overnight at 37°C/5% CO2. 5 days post transduction the transduced THP-1 cells were pelleted and resuspended in fresh RPMI supplemented with 10% fetal calf serum. 7 days post transduction, puromycin (Sigma) was added at 2 μg/ml and the selective media was refreshed every 2 days.

### Analysis of HCMV latency and reactivation

All latent infections in undifferentiated myeloid cells were validated by RTqPCR demonstrating a repression of IE gene expression and the presence of UL138 and the ability to reactivate virus using differentiation factors, such as PML, as described in the text and in previously published studies (Aslam et al., 2019; Poole et al., 2021).

## Results

### SERBP1 is upregulated during latency

**Figure 1:**
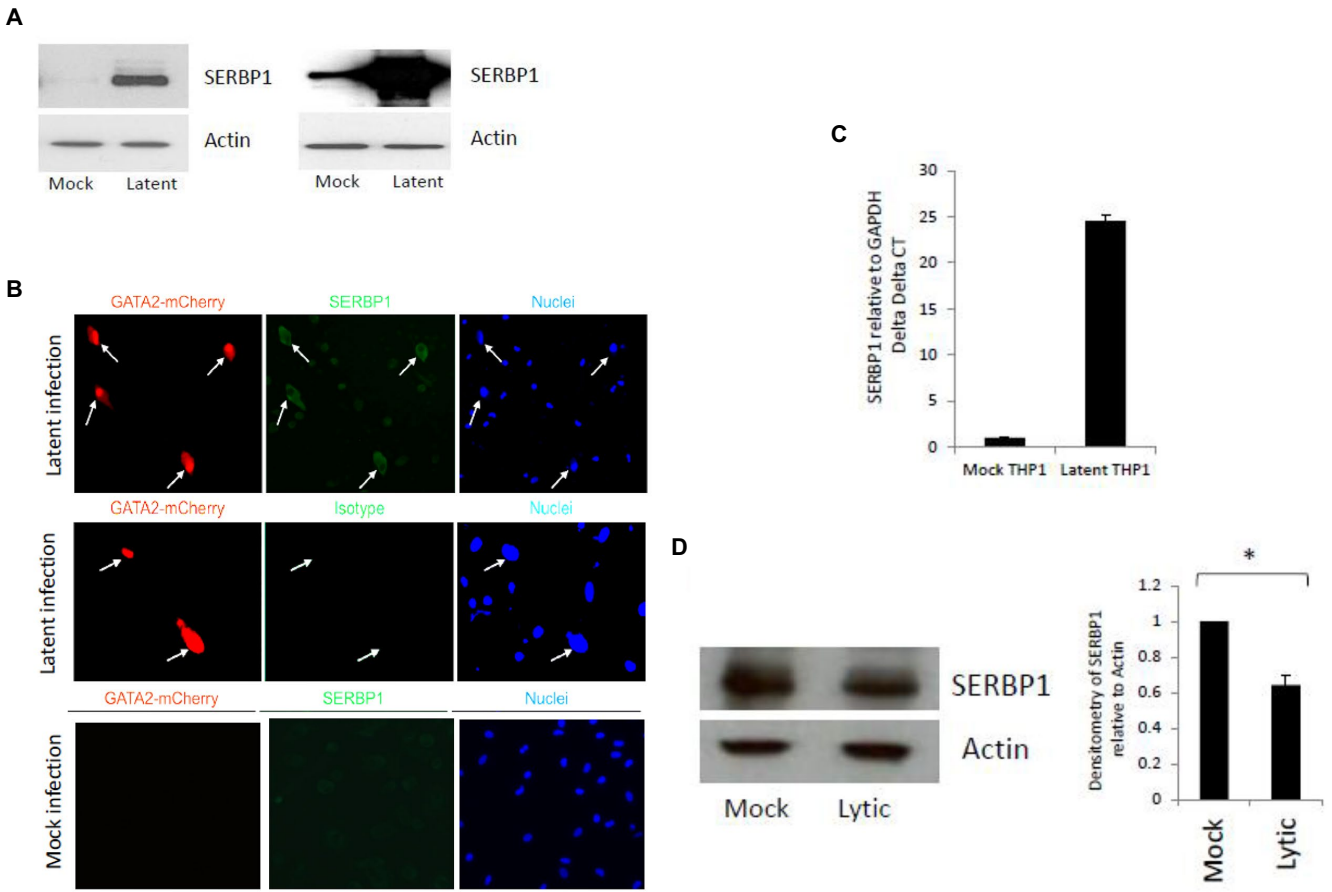
Validation of SERBP1 upregulation during HCMV latent and lytic infection. CD14+ cells were infected (latent) or uninfected (mock) with TB40E-GATA2mCherry for 6 days and then the latently infected cells were sorted by FACS before western blotting and two independent experiments are shown in the left and right hand panels **(A)**. Alternatively, CD14+ primary cells were either mock or latently infected with TB40E GATA2mCherry before staining for SERBP1 using a rabbit SERBP1 primary antibody followed by FITC anti-rabbit secondary antibody (top and bottom panels, SERBP1) or the equivalent isotype control (middle panel, isotype) and counter stained with Hoechst 33342 (nuclei; **B**). Finally, the cells were harvested for RNA analysis and RTqPCR carried out for GAPDH and SERBP1 **(C)**. These results represent triplicate technical repeats from two different donors. Additionally, HFFF cells were infected for 72 h before harvesting for western blot analysis of GAPDH and SERBP1 and densitometry carried out **(D)**. * = *p* value of <0.01.

In a previous proteome analysis using unbiased Tandem Mass Tag technology approach (Aslam et al., 2019) we identified that SERBP1 was one of the most highly upregulated proteins in a primary monocyte model of experimental HCMV latency (a reanalysis of this data is shown in Supplementary Figures 1A,B). Consequently, we validated this upregulation of SERBP1 during HCMV latency using a TB40E-SV40GFP virus, which allows the sorting of latently infected CD14+ monocytes expressing GFP, by western blot analysis. Figure 1A shows that latent infection of CD14+ monocytes results in a substantial increase in SERBP1 levels compared to mock infected cells. Two independent experiments are shown which both demonstrate that the SERBP1 upregulation is very high during latency and that it can be difficult to detect the WT SERBP1 due to such high levels of SERBP1.This increase in SERBP1 was also confirmed in infected cells by indirect immunofluorescence (IF) using a TB40E-GATA2mCherry virus which expresses mCherry under the control of the GATA2 promoter, a promoter known to be active in myeloid cells. Figure 1B shows that when latently infected CD14+ monocytes (red) were stained for SERBP1 (green) there was an upregulation of SERBP1 in the latently infected cells compared to uninfected bystander cells (Figure 1B top panel) which was not observed in mock infected cells (Figure 1B bottom panel). To test whether the SERBP1 protein was upregulated as a consequence of mRNA upregulation, levels of SERBP1 RNA were analyzed by RTqPCR. Figure 1C shows that SERBP1 mRNA was upregulated during HCMV latency, which is consistent with the observed protein upregulation. We next addressed whether SERBP1 was also upregulated during lytic infection, to determine whether SERBP1 upregulation was just a consequence of any HCMV infection. Figure 1D shows that SERBP1 protein expression was downregulated during lytic HCMV infection in fibroblasts. These data are consistent with the previously published screen of proteomic changes during HCMV lytic infection (Weekes et al., 2014). Consequently, the upregulation of SERBP1 in latently infected cells was robust and validated using two different analyses and two different recombinant HCMVs and this contrasts the observations for lytic infection.

### SERBP1 is required for IE repression during latency

**Figure 2:**
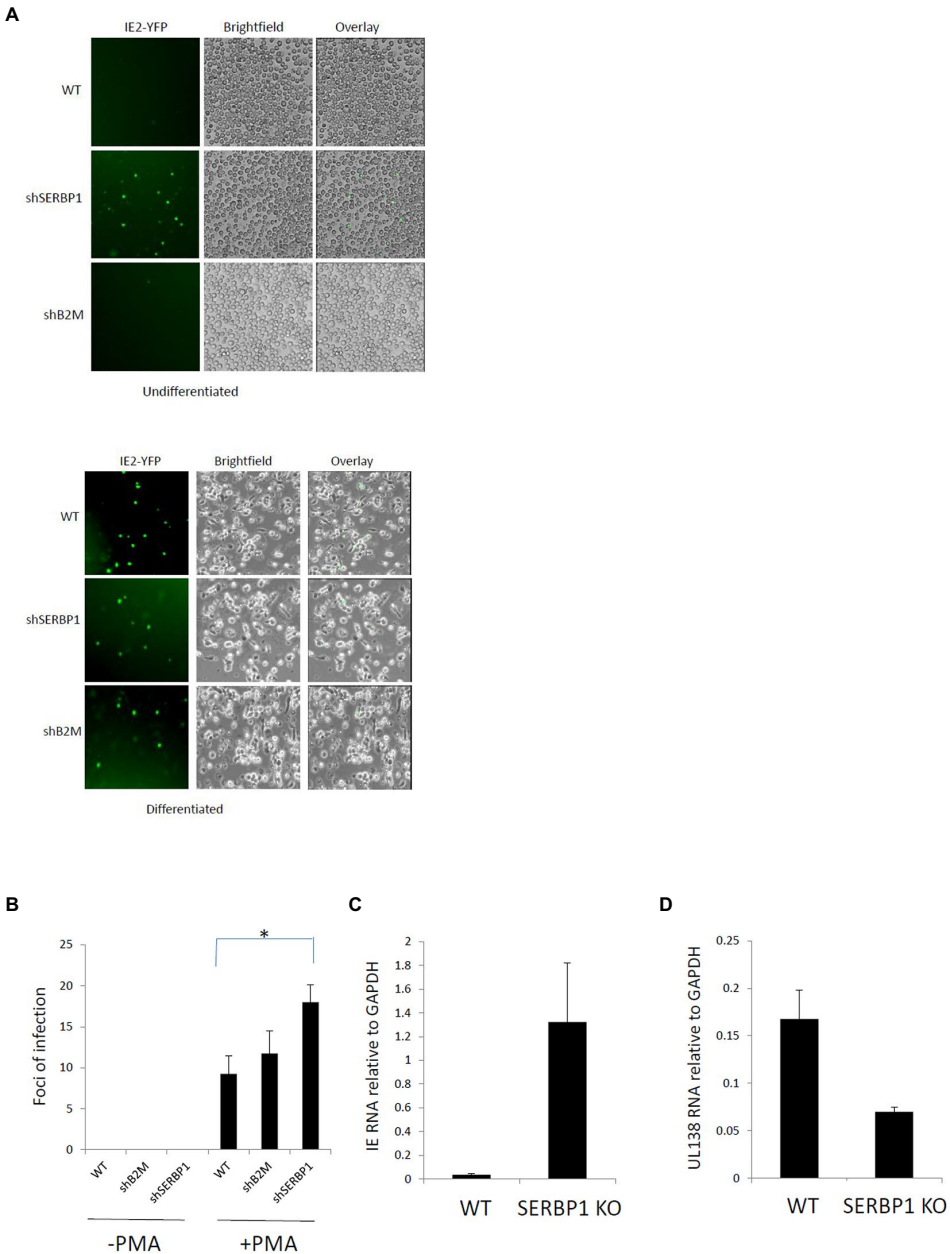
SERBP1 is required for repression of the MIEP during latency. WT, shB2M or shSERBP1 THP1 cells were infected with TB40E-IE2YFP for 4 days and then either reactivated with PMA or not before either analyzing by microscopy directly **(A)** or supernatants transferred to fibroblasts and foci of infection counted to detect productive virus reactivation **(B)** or harvesting cells for viral RNAs IE exon2/3 **(C)** and UL138 **(D)**. Data represent triplicate technical data from two independent experiments with standard deviation shown and the student’s *t*-test used for statistical analysis (* represents a *p-*value of <0.01).

To analyze the significance of this upregulation of SERBP1 during HCMV latency, we generated SERBP1 knock-down (KD) THP1 cells (Supplementary Figure 2) using shRNA technology cells; THP1 cells are a myelomonocytic cell line that has been routinely used as a model of HCMV latent (Poole et al., 2021). These cells were validated for the levels of SERBP1 gene expression which showed an 87% knockdown in these cells as shown by western blotting and densitometry (Supplementary Figures 2A,B). These cells were then infected with recombinant TB40E HCMV carrying an IE2YFP gene cassette which marks lytically infected cells, but not latently infected cells, with YFP (Straschewski et al., 2010). Figure 2A shows that, as expected, undifferentiated wild type (WT) THP1 cells or undifferentiated control THP1 cells in which Beta-2 microglobulin (B2M) was targeted using shRNA technology (removal of B2M has previously been shown to have no impact on the ability of undifferentiated cell types to establish HCMV latency (Poole et al., 2021)) do not express IE2 unless they are differentiated to a permissive macrophage-like phenotype. In contrast, undifferentiated SERBP1 KD THP1 cells failed to repress IE gene expression and express high levels of IE2YFP. All cells tested (WT, B2M or SERBP1 KD cells) express IE after differentiation, as expected. While expression of IE clearly occurred in the SERBP1 KD cells these cells did not progress through full lytic cycle as no virus production was observed upon their co-culture with indicator fibroblasts (Figure 2B, left panel). However, if these cells were differentiated, virus production was observed, as expected (Figure 2B, right panel). Consistent with this, WT THP1 cells infected with HCMV showed low levels of IE expression concomitant with good levels of UL138 expression (consistent with a latent infection (Poole et al., 2014)) whereas infected SERBP1 KD THP1 cells expressed high levels of IE exon2/3 RNA (Figures 2C,D). Taken together, these data shows that SERBP1 is required for the repression of IE gene expression in latently infected THP1 cells.

### SERBP1 mediates recruitment of both Kap1 and CHD3 to the HCMV genome during latency

**Figure 3:**
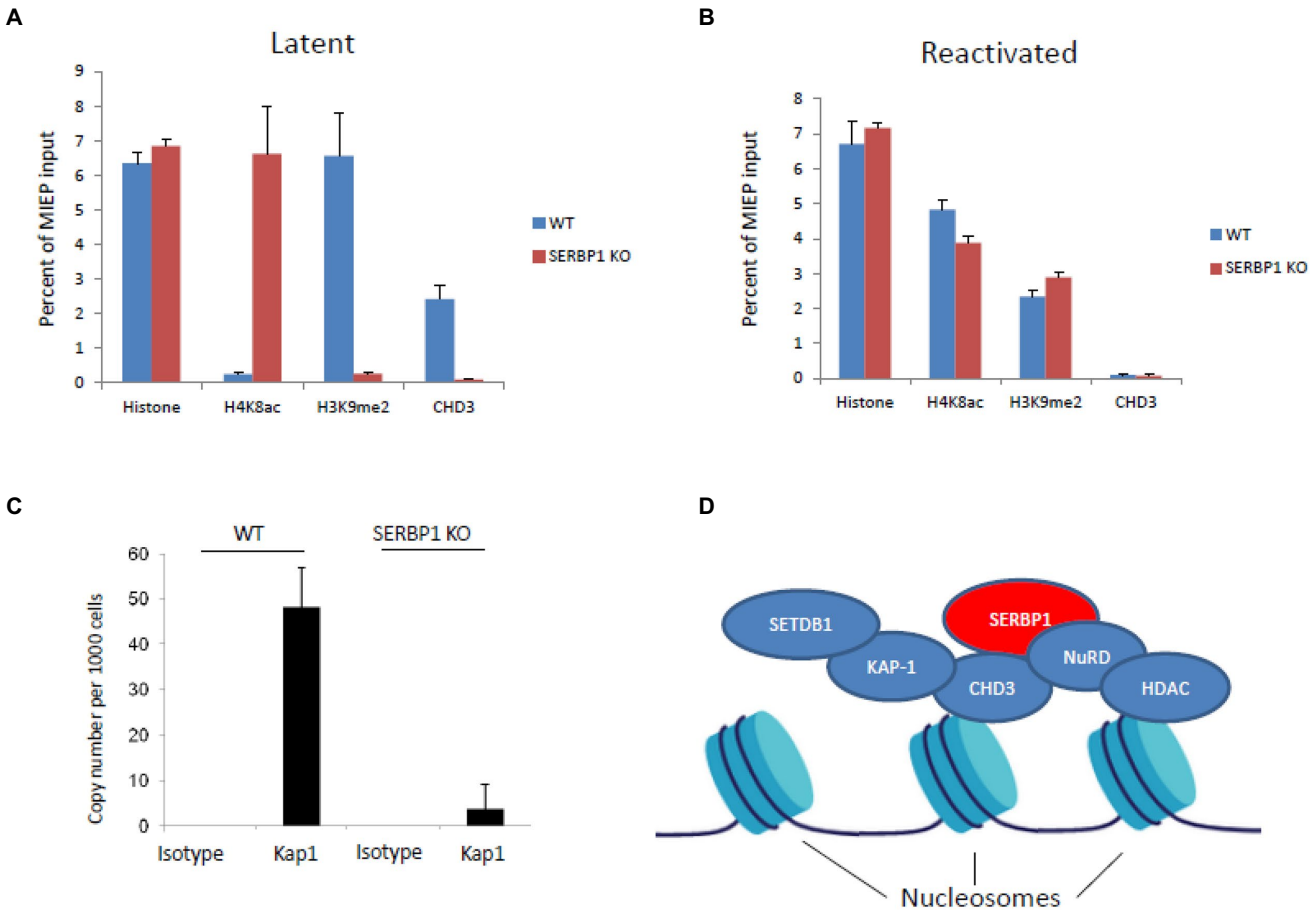
SERBP1 can recruit repressive proteins CHD3 and KAP1 to the viral genome. WT or shSERBP1 THP1 cells were either infected or left uninfected with TB40E-IE2YFP for 4 days before harvesting for ChIP analysis for histone H3 (“histone”) or activated (“H4K8ac”)or repressive (“H3K9me2”) histone markers or CHD3 during latency (latent, **A**) or reactivation (reactivated, **B**). Graphs represent standard deviation about the mean from two independent experiments with triplicate technical repeats. WT or shSERBP1 THP1 cells were infected with TB40E for 4 days before fixing in 1% PFA and then harvesting for ChIP analysis. DNA was then analyzed by ChIP for the presence of KAP1 and the numbers of genomes were quantified by droplet digital PCR using a gB probe **(C)**. Potential schematic shows how SERBP1 may act as a scaffold to form a repressive complex *via* CHD3 and HDAC (which are known to associate with histone proteins) to recruit KAP1 where SETDB1 can mediate methylation and the NuRD complex with HDAC proteins can mediate histone deacetylation **(D)**.

Since SERBP1 is known to bind to CHD3, which can associate with Kap1 and the NuRD complex, we tested whether Kap1 and CHD3 were able to associate with the viral genome in the absence of SERBP1. Figure 3A shows that, as expected, there is little difference in total histone association with the viral MIEP on infection of WT THP1 or SERBP1 KD THP1 cells when using an antibody for histone H3 (“histone”). Similarly, as expected, in infected WT THP1 cells the viral MIEP is associated with low levels of activatory acetylated histone marks (H4K8ac) and high levels of repressive methylated histone marks (H3K9me)—consistent with suppression of the MIEP and a latent infection. In contrast, in the absence of SERBP1, there is a profound increase in acetylated histone marks (H4K8ac) and a decrease in methylated histone marks (H3K9me) consistent with MIEP transcriptional activity. Importantly, CHD3 can only be found associated with the viral MIEP in the presence of SERBP1—removal of SERBP1 prevents CHD3 association with the promoter. In contrast, PMA induced reactivation of both infected WT and SERBP2 KD cells results in association of the MIEP with activatory histone marks (H4K8ac), and a reduction of repressive histone marks (H3K9me), as expected, with no association with CHD3 (Figure 3B). These data suggest that CHD3 association with the MIEP is required for efficient MIEP repression during latency and that removal of SERBP1 prevents association of CHD3 to the MIEP.

As KAP1 binding to the viral genome had been shown to be involved in MIEP repression during latent infection of primary myeloid cells (Rauwel et al., 2015), and because Kap1-associated protein CHD3 is known to bind SERBP1 (Lemos et al., 2003), we assessed the binding of KAP1 to the viral genome in latently infected THP1 cells in the presence or absence of SERBP1. Figure 3C shows that in the absence of SERBP1, Kap1 no longer associates with the HCMV genomes in infected THP1 cells in these cells in which IE gene expression but not full virus reactivation has occurred. These data suggest that SERBP1 acts as a recruitment factor to facilitate Kap1 association with the viral genome during latent infection.

## Discussion

The regulation of the MIEP during latent and lytic infection is complex and controlled by multiple mechanisms involving a plethora of cellular and viral factors. It is clear, though, that during latent infection of undifferentiated myeloid cells the HCMV MIEP is associated with repressive histone marks which are replaced by activatory histone marks as the cell differentiates and reactivation of major IE gene expression is initiated. To date, cellular factors involved in MIEP suppression during latency have identified specific cellular transcription factors that bind to known transcription factor binding sites in the MIEP (Bain et al., 2003; Martinez et al., 2014; Elder et al., 2021; Poole et al., 2021). In this paper, we have identified SERBP1 as an additional factor involved in control of the MIEP during latency.

The data presented do not exclude the possibility that there are other mechanisms by which SERBP1 functions to regulate the MIEP, for example, it may be that these effects are mediated by the known RNA binding functions of SERBP1 which occur in the cytoplasm. Our data show that SERBP1 is located in the cytoplasm and so it is quite likely that it plays a role which perhaps affects CHD3 and KAP1. Additionally, SERBP1 is known to play a role in the regulation of methionine levels which may also be important for generating a repressive chromatin structure (Kosti et al., 2020) and these possibilities need to be investigated in future work. However, given the observed effects on CHD3 and KAP1 shown in this paper, it is does suggest that SERBP1 could act as a “scaffold protein” to recruit repressive accessory factors CHD3 (Lemos et al., 2003) and Kap1 to help to prevent activation of IE gene expression during HCMV latency. A potential model is outlined in Figure 3D where the histone-associating proteins CHD3 (Tencer et al., 2017) and HDAC are shown to be associated with nucleosomes. SERBP1 could mediate CHD3 and, in turn Kap1, association with the viral genome. These proteins being associated with the genome would support a repressive environment because Kap1 mediates methylation of histones *via* SETDB1 and HDACs lead to histone deacetylation.

While we have not addressed the trigger for SERBP1 upregulation during latency in this manuscript, it is interesting to note that the transcription factor YY1 can drive the expression of SMHG8 which, in turn, can lead to an upregulation of SERBP1 (Shan et al., 2022). Since it has been previously published that YY1 is critical for the maintenance of HCMV latency (Poole et al., 2021) it is possible that this contributes to the upregulation of SERBP1 during latency. SERBP1 is known to shuttle between the cytoplasm and the nucleus (Lee et al., 2012, 2014) and certainly in lytic infection has been observed to be located in both locations (Weekes et al., 2014). Our data suggests that SERBP1 is predominatly cytoplasmic and it is possible that SERBP1 is mediating effects *via* a cytoplasmic route and this is, as stated above, is something that should be investigated in future studies. However, given the effects on CHD3 and KAP1, our observations here are consistent with it acting as a scaffold protein to recruit repressive complexes, involving CHD3 and KAP1, to the viral genome in order to help maintain the viral genome in a repressive chromatin structure thereby aiding maintenance of latency. Interestingly, as well as HCMV, KAP1 has also been shown to be required for KSHV latency where the KSHV LANA gene product has been proposed to mediate recruitment of Kap1 to the KSHV genome (Sun et al., 2014). Similarly, depletion of CHD3 in during Herpes Simplex Virus (HSV-1) infection breaks latency in an HSV-1 latency model (Arbuckle and Kristie, 2014). It would be of interest to determine whether these other herpesviruses also use SERBP1 as a scaffold to recruit both KAP1 and CHD3 to their genomes to initiate and maintain a repressive chromatin structure during latent infection.

## Data availability statement

The original contributions presented in the study are included in the article/Supplementary material, further inquiries can be directed to the corresponding author.

## Ethics statement

All research describing studies on primary human material with HCMV were assessed and approved by the Cambridge Local Research Ethics committee. Informed consent was received from blood donors with the Cambridge Local Research Ethics committee and the Cambridge Internal Review Board. Cells were harvested from healthy adult donors, and the decision to use tissue was not affected by gender and age, as this was not important to the studies performed. The patients/participants provided their written informed consent to participate in this study.

## Author contributions

EP performed experiments and wrote the manuscript. JS edited the manuscript. All authors contributed to the article and approved the submitted version.

## Funding

This work was funded by the British Medical Research Council, grant number MR/S00081X/1/MRC.

## Conflict of interest

The authors declare that the research was conducted in the absence of any commercial or financial relationships that could be construed as a potential conflict of interest.

The reviewer MN declared a past collaboration with one of the authors EP to the handling editor.

## Publisher’s note

All claims expressed in this article are solely those of the authors and do not necessarily represent those of their affiliated organizations, or those of the publisher, the editors and the reviewers. Any product that may be evaluated in this article, or claim that may be made by its manufacturer, is not guaranteed or endorsed by the publisher.

## Supplementary material

SUPPLEMENTARY FIGURE 1 SERBP1 is upregulated during HCMV latency. Primary CD14 monocytes were infected with TB40E-SV40GFP. The green cells were then sorted by FACS before full proteomic screen analysis as previously described (Aslam et al., 2019). The proteins identified with the highest number of peptides are shown from two independent screens (A). The data for all the hits are plotted on a graph (B) where each + represents a protein and all proteins are represented along the x axis in alphabetical order (proteins).

SUPPLEMENTARY FIGURE 2 Validation of SERBP1 knock down cells. THP1 shSERBP1 cells were western blotted for SERBP1 alongside WT THP1 cells **(A)** and the levels of knock down determined by densitometry with standard deviations shown and the student’s T test statistical analysis to determine the statistical significance (*** represents a P value >0.0001) **(B)**.
