# Supplementary material for: Latency-associated upregulation of SERBP1 is important for the recruitment of transcriptional repressors to the viral major immediate early promoter of human cytomegalovirus during latent carriage
Source: Front Microbiol. 2022 Nov 24;13:999290. doi: 10.3389/fmicb.2022.999290 (PMC9729347; doi:10.3389/fmicb.2022.999290)
Supplement: SUPPLEMENTARY FIGURE 1 — SERBP1 is upregulated during HCMV latency. Primary CD14 monocytes were infected with TB40E-SV40GFP. The green cells were then sorted by FACS before full proteomic screen analysis as previously described (Aslam et al., 2019). The proteins identified with the highest number of peptides are shown from two independent screens (A). The data for all the hits are plotted on a graph (B) where each + represents a protein and all proteins are represented along the x axis in alphabetical order (proteins). [file Presentation_1.pdf]

Supplementary Figure 1

1A

Accession	Description		Unique peptides	+/-
P14317	Hematopoietic lineage cell-specific protein	HCLS1	7	51.125
Q8NC51	Plasminogen activator inhibitor 1 RNA-binding protein	SERBP1	6	65.25
P05120	Plasminogen activator inhibitor 2	SERPINB2	4	2.415902
P06702	Protein S100-A9	S100A9	11	-18.089
P05109	Protein S100-A8	S100A8	14	-18.1589

Accession	Description		Unique peptides	+/-
P14317	Hematopoietic lineage cell-specific protein	HCLS1	22	11.49259
Q8NC51	Plasminogen activator inhibitor 1 RNA-binding protein	SERBP1	14	10.39138
P05120	Plasminogen activator inhibitor 2	SERPINB2	12	23.11794
P05109	Protein S100-A8	S100A8	16	-19.2139
P06702	Protein S100-A9	S100A9	13	-17.6611

1B

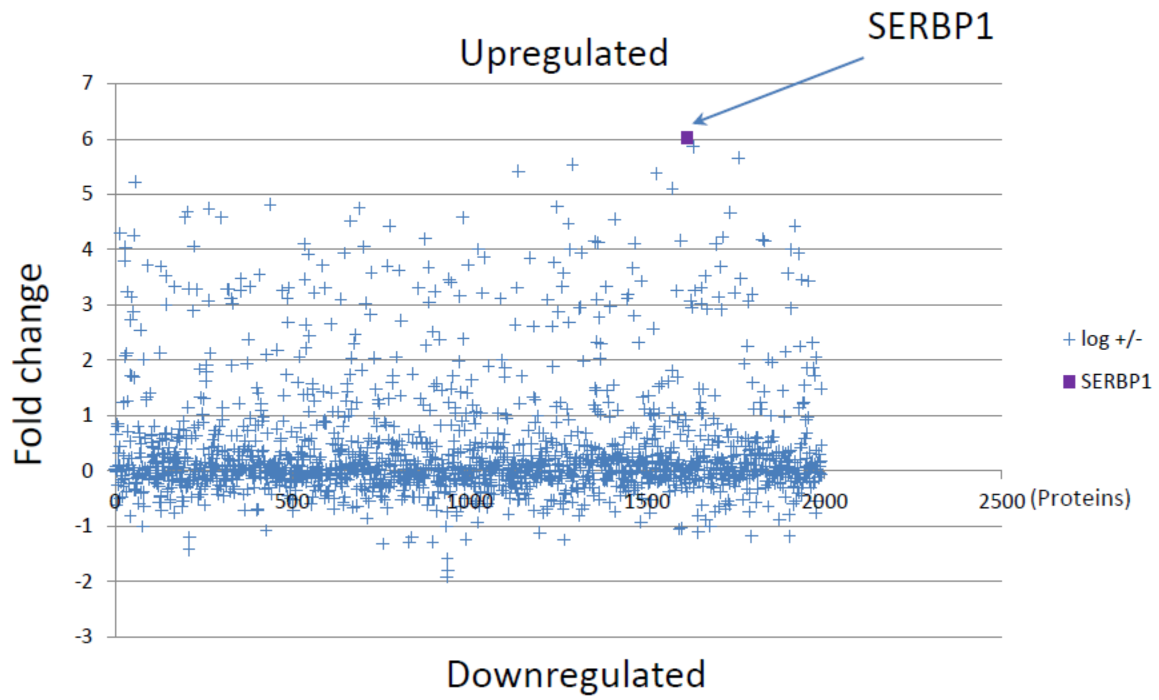

Supplementary figure 2

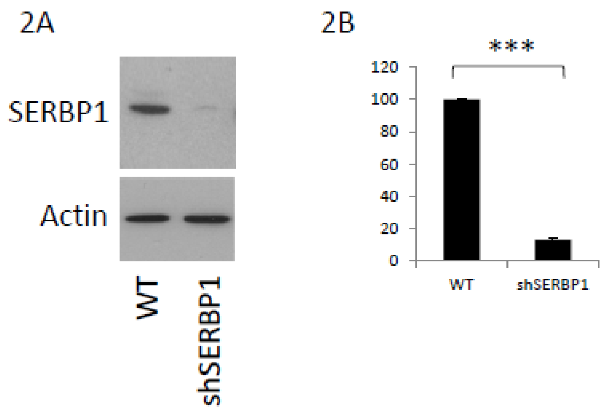

Supplementary Figure 1

SERBP1 is upregulated during HCMV latency. Primary CD14 monocytes were infected with TB40E-SV40GFP. The green cells were then sorted by FACS before full proteomic screen analysis as previously described (13). The proteins identified with the highest number of peptides are shown from two independent screens (A). The data for all the hits are plotted on a graph (B) where each + represents a protein and all proteins are represented along the x axis in alphabetical order (proteins).

Supplementary Figure 2

Validation of SERBP1 knock down cells. THP1 shSERBP1 cells were western blotted for SERBP1 alongside WT THP1 cells (A) and the levels of knock down determined by densitometry with standard deviations shown and the student's T test statistical analysis to determine the statistical significance (*** represents a P value >0.0001) (B).
